# Tetra­kis(1,1,1-trifluoro­acetyl­acetonato-κ^2^ *O*,*O*′)hafnium(IV) toluene disolvate

**DOI:** 10.1107/S1600536808015237

**Published:** 2008-05-24

**Authors:** J. Augustinus Viljoen, Alfred Muller, Andreas Roodt

**Affiliations:** a Department of Chemistry, University of the Free State, PO Box 339, Bloemfontein 9300, South Africa

## Abstract

In the title compound, [Hf(C_5_H_4_F_3_O_2_)_4_]·2C_7_H_8_, the Hf^IV^ atom, lying on a twofold rotation axis, is coordinated by eight O atoms from four 1,1,1-trifluoro­acetyl­acetonate ligands with an average Hf—O distance of 2.173 (1) Å and O—Hf—O bite angles of 75.69 (5) and 75.54 (5)°. The coordination polyhedron shows a slightly distorted Archimedean square antiprismatic geometry. The asymmetric unit contains a toluene solvent mol­ecule. The crystal structure involves C—H⋯.F hydrogen bonds.

## Related literature

For the triclinic polymorph of the title compound, see: Zherikova *et al.* (2005 ▶). For related literature on hafnium β-diketone complexes, see: Chattoraj *et al.* (1968 ▶). For the isomorphous zirconium complex, see: Steyn *et al.* (2008 ▶). For a description of the Cambridge Structural Database, see: Allen (2002 ▶).
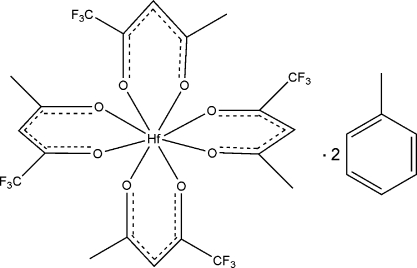


## Experimental

### 

#### Crystal data

[Hf(C_5_H_4_F_3_O_2_)_4_]·2C_7_H_8_
*M* *_r_* = 975.09
Monoclinic, 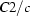

*a* = 22.4983 (15) Å
*b* = 8.0642 (5) Å
*c* = 22.712 (2) Å
β = 118.211 (2)°
*V* = 3631.2 (5) Å^3^
*Z* = 4
Mo *K*α radiation
μ = 2.98 mm^−1^
*T* = 100 (2) K
0.33 × 0.25 × 0.22 mm

#### Data collection

Bruker Kappa APEXII diffractometer
Absorption correction: multi-scan (*SADABS*; Bruker, 2001 ▶) *T*_min_ = 0.439, *T*_max_ = 0.560 (expected range = 0.407–0.519)
18332 measured reflections
4518 independent reflections
4256 reflections with *I* > 2σ(*I*)
*R*_int_ = 0.023

#### Refinement

*R*[*F* ^2^ > 2σ(*F* ^2^)] = 0.018
*wR*(*F* ^2^) = 0.042
*S* = 1.07
4518 reflections
252 parameters
H-atom parameters constrained
Δρ_max_ = 1.12 e Å^−3^
Δρ_min_ = −0.91 e Å^−3^

### 

Data collection: *APEX2* (Bruker, 2007 ▶); cell refinement: *SAINT-Plus* (Bruker, 2007 ▶); data reduction: *SAINT-Plus*; program(s) used to solve structure: *SIR97* (Altomare *et al.*, 1999 ▶); program(s) used to refine structure: *SHELXL97* (Sheldrick, 2008 ▶); molecular graphics: *DIAMOND* (Brandenburg & Putz, 1999 ▶); software used to prepare material for publication: *WinGX* (Farrugia, 1999 ▶).

## Supplementary Material

Crystal structure: contains datablocks global, I. DOI: 10.1107/S1600536808015237/hy2134sup1.cif

Structure factors: contains datablocks I. DOI: 10.1107/S1600536808015237/hy2134Isup2.hkl

Additional supplementary materials:  crystallographic information; 3D view; checkCIF report

## Figures and Tables

**Table:** 

Hf—O2	2.1527 (13)
Hf—O4	2.1571 (13)
Hf—O1	2.1861 (13)
Hf—O3	2.1933 (13)

**Table:** 

O2—Hf—O2^i^	141.66 (7)
O2—Hf—O4	80.96 (5)
O2^i^—Hf—O4	111.77 (5)
O4—Hf—O4^i^	142.02 (7)
O2—Hf—O1^i^	141.35 (5)
O4—Hf—O1^i^	72.52 (5)
O2—Hf—O1	75.69 (5)
O4—Hf—O1	76.79 (5)
O1^i^—Hf—O1	71.28 (7)
O2—Hf—O3^i^	72.21 (5)
O4—Hf—O3^i^	141.11 (5)
O1—Hf—O3^i^	121.11 (5)
O2—Hf—O3	76.82 (5)
O4—Hf—O3	75.54 (5)
O1—Hf—O3	143.48 (5)
O3^i^—Hf—O3	71.35 (7)

Symmetry code: (i) 
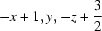
.

**Table 2:** Hydrogen-bond geometry (Å, °)

*D*—H⋯*A*	*D*—H	H⋯*A*	*D*⋯*A*	*D*—H⋯*A*
C3—H3⋯F2	0.93	2.37	2.712 (2)	102
C8—H8⋯F5	0.93	2.37	2.721 (2)	102

**Fig. 1.**
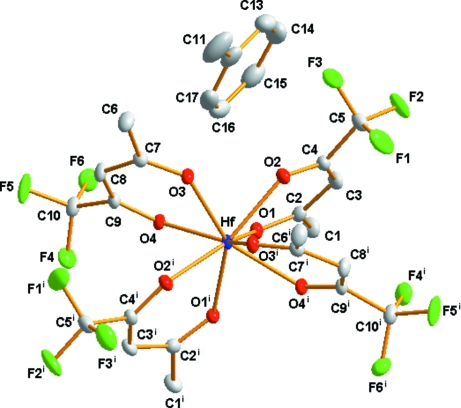
Structure of the title compound. Displacement ellipsoids are drawn at the 30 % probability level. H atoms have been omitted for clarity.

**Fig. 2.**
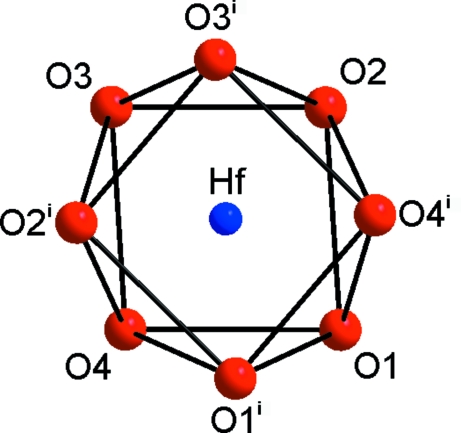
Slightly distorted Archimedean antiprism coordination polyhedron surrounding Hf atom.

**Fig. 3.**
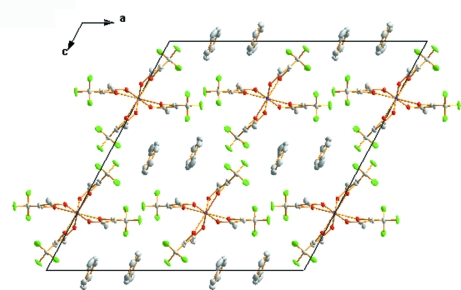
Packing diagram of the title compound along the b-axis showing π-stacking of toluene molecule pairs. H atoms have been omitted for clarity.

## supplementary crystallographic information

### Comment

This study was done as part of ongoing research in our group to investigate reactions of O,O'- and O,N-bidentate ligands with hafnium(IV) and zirconium(IV). The total separation of zircon ore (ZrSiO_4_) is important to have materials viable for nuclear applications. Previous work on hafnium(IV) complexes with β-diketone was done to determine their thermal decomposition to yield metal oxides (Chattoraj *et al.*,  1968). Hafnium β-diketonates are also promising precursor materials for producing metal oxide films, providing the possibility to manufacture technologically important coatings (Zherikova *et al.*,  2005).

The title compound crystallizes as the monoclinic polymorph (*C*2/*c*, *Z* = 4) (Fig. 1) with two toluene solvent molecules. The triclinic polymorph earlier reported by Zherikova *et al.* (2005) contains no solvent molecules and cannot be superimposed with the title compound due to differences in metal coordination modes. An isomorphous zirconium complex has been reported by Steyn *et al.* (2008). The Hf^IV^ atom in the title compound is situated on a twofold rotation axis, with four β-diketonate ligands, 1,1,1-trifluoroacetylacetonate (tfaa), coordinating to the Hf^IV^ atom adopting an Archimedean antiprism coordination geometry (Fig. 2). The Hf—O bond lengths vary from 2.1527 (13) Å to 2.1933 (13) Å, with the average Hf—O distance being 2.173 (1) Å. The O—Hf—O bite angles are 75.69 (5)° and 75.54 (5)° (Table 1). This average bond distance is somewhat larger than the average of 2.156 Å obtained from the Cambridge Structural Database (Allen, 2002) (data extracted from 19 hits, yielding 45 observations ranging from 2.039 to 2.248 Å). Pairs of toluene molecules are π-stacked (interplanar distance = 3.65 (1) Å, centroid–centroid distance = 4.92 (1) Å) in channels formed by the metal complex moieties parallel to the *b*-axis (Fig. 3). The preferred CF_3_-group conformation is probably due to weak C—H···F interactions (Table 2).

### Experimental

Chemicals were purchased from Sigma and Aldrich and used as received except for toluene, which was dried by passage over alumina. Syntheses were performed using modified Schlenk conditions. The ligand salt (Natfaa) was prepared by adding Htfaa (6.05 ml, 50 mmol) dropwise to NaOH (2.02 g, 50 mmol) over a period of 3 minutes. The resulting solids were washed with toluene and dried *in vacuo*. Natfaa (0.459 g, 2.6 mmol) was added to a suspension of HfCl_4_ (0.207 g, 0.65 mmol) in toluene (10 ml). Dissolution gave a slightly yellow solution after 10 min. After refluxing for *ca* 20 h the crude product was filtered and washed with toluene. The filtrate was slowly recrystallized at 253 K at near quantitative yield. Spectroscopy data: ^19^F {H} NMR (C_6_D_6_; 564.77 MHz): -75.49 p.p.m.; IR (ATR): ν(CO) 1533 cm^-1^.

### Refinement

H atoms were positioned geometrically and refined as riding atoms, with C—H = 0.93 (aromatic) and 0.96 Å (methyl) and with *U*_iso_(H) = 1.2*U*_eq_(C) for aromatic and 1.5*U*_eq_(C) for methyl groups. Torsion angles for methyl H atoms were refined from electron density. The highest residual electron density lies within 1.0 Å from the Hf atom.

### Crystal data

**Table:**

[Hf(C_5_H_4_F_3_O_2_)_4_]·2C_7_H_8_	*F*_000_ = 1920
*M**_r_* = 975.09	*D*_x_ = 1.784 Mg m^−^^3^
Monoclinic, *C*2/*c*	Mo *K*α radiation λ = 0.71073 Å
Hall symbol: -C 2yc	Cell parameters from 6211 reflections
*a* = 22.4983 (15) Å	θ = 2.7–28.3º
*b* = 8.0642 (5) Å	µ = 2.98 mm^−^^1^
*c* = 22.712 (2) Å	*T* = 100 (2) K
β = 118.211 (2)º	Block, colourless
*V* = 3631.2 (5) Å^3^	0.33 × 0.25 × 0.22 mm
*Z* = 4	

### Data collection

**Table:**

Bruker X8 APEXII 4K KappaCCD diffractometer	4518 independent reflections
Monochromator: graphite	4256 reflections with *I* > 2σ(*I*)
Detector resolution: 8.4 pixels mm^-1^	*R*_int_ = 0.023
*T* = 100(2) K	θ_max_ = 28.4º
φ and ω scans	θ_min_ = 2.0º
Absorption correction: multi-scan(SADABS; Bruker, 2001)	*h* = −30→26
*T*_min_ = 0.439, *T*_max_ = 0.560	*k* = −10→10
18332 measured reflections	*l* = −29→30

### Refinement

**Table:**

Refinement on *F*^2^	H-atom parameters constrained
Least-squares matrix: full	*w* = 1/[σ^2^(*F*_o_^2^) + (0.0173*P*)^2^ + 5.3839*P*] where *P* = (*F*_o_^2^ + 2*F*_c_^2^)/3
*R*[*F*^2^ > 2σ(*F*^2^)] = 0.019	(Δ/σ)_max_ = 0.002
*wR*(*F*^2^) = 0.042	Δρ_max_ = 1.12 e Å^−^^3^
*S* = 1.07	Δρ_min_ = −0.91 e Å^−^^3^
4518 reflections	Extinction correction: none
252 parameters	

### Special details

**Table:**

Experimental. The intensity data was collected on a Bruker X8 Apex II 4 K Kappa CCD diffractometer using an exposure time of 20 s/frame. A total of 1897 frames were collected with a frame width of 0.5° covering up to θ = 28.35° with 99.8% completeness accomplished.
Geometry. All e.s.d.'s (except the e.s.d. in the dihedral angle between two l.s. planes) are estimated using the full covariance matrix. The cell e.s.d.'s are taken into account individually in the estimation of e.s.d.'s in distances, angles and torsion angles; correlations between e.s.d.'s in cell parameters are only used when they are defined by crystal symmetry. An approximate (isotropic) treatment of cell e.s.d.'s is used for estimating e.s.d.'s involving l.s. planes.

### Fractional atomic coordinates and isotropic or equivalent isotropic displacement parameters (Å^2^)

**Table:**

	*x*	*y*	*z*	*U*_iso_*/*U*_eq_	
Hf	0.5	0.165295 (14)	0.75	0.01071 (4)	
O1	0.51936 (7)	−0.05501 (17)	0.81255 (6)	0.0145 (3)	
O2	0.49393 (7)	0.25296 (17)	0.83653 (6)	0.0150 (3)	
C1	0.50749 (11)	−0.2808 (3)	0.87229 (11)	0.0220 (4)	
H1A	0.508	−0.3448	0.8369	0.033*	
H1B	0.4696	−0.3133	0.8783	0.033*	
H1C	0.5485	−0.2998	0.9129	0.033*	
C2	0.50184 (10)	−0.1008 (3)	0.85482 (9)	0.0156 (4)	
C3	0.47953 (10)	0.0117 (3)	0.88823 (10)	0.0184 (4)	
H3	0.4644	−0.0284	0.9172	0.022*	
C4	0.48009 (10)	0.1778 (3)	0.87820 (9)	0.0154 (4)	
C5	0.46372 (11)	0.2975 (3)	0.92078 (10)	0.0207 (4)	
F1	0.40902 (8)	0.3862 (2)	0.88355 (7)	0.0409 (4)	
F2	0.45382 (8)	0.21987 (18)	0.96725 (7)	0.0343 (3)	
F3	0.51363 (7)	0.40485 (17)	0.95280 (7)	0.0316 (3)	
O3	0.43603 (7)	0.38624 (17)	0.71276 (7)	0.0146 (3)	
O4	0.40009 (6)	0.07826 (17)	0.72464 (6)	0.0136 (3)	
C6	0.36205 (11)	0.6104 (3)	0.69597 (12)	0.0229 (4)	
H6A	0.4012	0.6754	0.7049	0.034*	
H6B	0.3434	0.6475	0.724	0.034*	
H6C	0.329	0.6231	0.6499	0.034*	
C7	0.38151 (10)	0.4318 (2)	0.71005 (9)	0.0158 (4)	
C8	0.33652 (10)	0.3194 (2)	0.71708 (10)	0.0175 (4)	
H8	0.2986	0.3595	0.7188	0.021*	
C9	0.34868 (10)	0.1536 (2)	0.72125 (9)	0.0148 (4)	
C10	0.29459 (10)	0.0341 (3)	0.71829 (11)	0.0197 (4)	
F4	0.31970 (6)	−0.07336 (16)	0.76889 (6)	0.0261 (3)	
F5	0.24293 (7)	0.11096 (17)	0.72015 (8)	0.0338 (3)	
F6	0.26890 (6)	−0.05534 (17)	0.66195 (6)	0.0274 (3)	
C11	0.65622 (14)	0.3966 (4)	0.96674 (17)	0.0550 (9)	
H11A	0.6943	0.427	0.9608	0.082*	
H11B	0.6541	0.4676	0.9996	0.082*	
H11C	0.6156	0.4084	0.9251	0.082*	
C12	0.66358 (11)	0.2194 (3)	0.98981 (12)	0.0313 (5)	
C13	0.66964 (12)	0.1760 (3)	1.05128 (12)	0.0336 (6)	
H13	0.6681	0.258	1.0793	0.04*	
C14	0.67800 (13)	0.0117 (4)	1.07158 (12)	0.0376 (6)	
H14	0.682	−0.0156	1.1131	0.045*	
C15	0.68046 (13)	−0.1112 (4)	1.03081 (14)	0.0384 (6)	
H15	0.6867	−0.2213	1.0446	0.046*	
C16	0.67360 (12)	−0.0688 (4)	0.96942 (14)	0.0406 (7)	
H16	0.6747	−0.1509	0.9412	0.049*	
C17	0.66513 (12)	0.0941 (4)	0.94935 (12)	0.0372 (6)	
H17	0.6603	0.1204	0.9075	0.045*	

### Atomic displacement parameters (Å^2^)

**Table:**

	*U*^11^	*U*^22^	*U*^33^	*U*^12^	*U*^13^	*U*^23^
Hf	0.01298 (6)	0.00766 (6)	0.01517 (6)	0	0.00966 (4)	0
O1	0.0157 (6)	0.0118 (7)	0.0192 (6)	0.0011 (5)	0.0108 (5)	0.0027 (5)
O2	0.0207 (7)	0.0117 (7)	0.0170 (6)	−0.0013 (6)	0.0126 (6)	0.0003 (5)
C1	0.0315 (12)	0.0136 (10)	0.0271 (11)	0.0000 (9)	0.0190 (9)	0.0039 (8)
C2	0.0145 (9)	0.0143 (10)	0.0175 (9)	−0.0012 (8)	0.0071 (7)	0.0020 (8)
C3	0.0239 (10)	0.0171 (10)	0.0195 (9)	−0.0018 (8)	0.0148 (8)	0.0026 (8)
C4	0.0160 (9)	0.0166 (10)	0.0161 (8)	−0.0014 (8)	0.0097 (7)	−0.0013 (8)
C5	0.0300 (11)	0.0179 (11)	0.0211 (10)	0.0006 (8)	0.0179 (9)	0.0009 (8)
F1	0.0431 (9)	0.0495 (10)	0.0335 (8)	0.0248 (8)	0.0209 (7)	−0.0008 (7)
F2	0.0647 (10)	0.0234 (7)	0.0379 (8)	−0.0053 (7)	0.0433 (8)	−0.0026 (6)
F3	0.0485 (9)	0.0217 (7)	0.0361 (7)	−0.0106 (6)	0.0294 (7)	−0.0127 (6)
O3	0.0160 (7)	0.0110 (7)	0.0210 (7)	0.0019 (5)	0.0122 (6)	0.0026 (5)
O4	0.0136 (6)	0.0109 (7)	0.0194 (7)	0.0020 (5)	0.0104 (5)	0.0003 (5)
C6	0.0229 (11)	0.0131 (10)	0.0375 (12)	0.0034 (8)	0.0183 (10)	0.0040 (9)
C7	0.0187 (9)	0.0124 (10)	0.0183 (9)	0.0012 (8)	0.0104 (8)	0.0008 (7)
C8	0.0164 (9)	0.0131 (10)	0.0288 (10)	0.0017 (8)	0.0155 (8)	0.0005 (8)
C9	0.0147 (9)	0.0147 (10)	0.0186 (9)	0.0005 (8)	0.0109 (7)	0.0002 (8)
C10	0.0185 (10)	0.0143 (10)	0.0312 (11)	0.0008 (8)	0.0158 (9)	0.0002 (8)
F4	0.0291 (7)	0.0205 (7)	0.0348 (7)	−0.0030 (5)	0.0201 (6)	0.0060 (6)
F5	0.0273 (7)	0.0185 (7)	0.0731 (10)	0.0012 (6)	0.0382 (7)	−0.0005 (7)
F6	0.0228 (6)	0.0246 (7)	0.0335 (7)	−0.0089 (5)	0.0122 (6)	−0.0067 (6)
C11	0.0259 (14)	0.0496 (19)	0.067 (2)	−0.0055 (13)	0.0033 (13)	0.0194 (16)
C12	0.0156 (10)	0.0374 (14)	0.0306 (12)	−0.0050 (10)	0.0024 (9)	0.0040 (11)
C13	0.0294 (12)	0.0384 (15)	0.0324 (12)	−0.0058 (11)	0.0141 (10)	−0.0126 (11)
C14	0.0352 (13)	0.0514 (18)	0.0272 (12)	−0.0092 (13)	0.0154 (10)	0.0039 (12)
C15	0.0270 (13)	0.0302 (14)	0.0523 (16)	−0.0051 (11)	0.0141 (12)	−0.0008 (12)
C16	0.0235 (12)	0.0549 (19)	0.0425 (15)	−0.0074 (12)	0.0150 (11)	−0.0250 (14)
C17	0.0229 (12)	0.065 (2)	0.0222 (11)	−0.0077 (12)	0.0094 (10)	−0.0013 (12)

### Geometric parameters (Å, °)

**Table:**

Hf—O2	2.1527 (13)	C6—H6B	0.96
Hf—O2^i^	2.1527 (13)	C6—H6C	0.96
Hf—O4	2.1571 (13)	C7—C8	1.423 (3)
Hf—O4^i^	2.1571 (13)	C8—C9	1.359 (3)
Hf—O1^i^	2.1861 (13)	C8—H8	0.93
Hf—O1	2.1861 (13)	C9—C10	1.529 (3)
Hf—O3^i^	2.1933 (14)	C10—F4	1.333 (2)
Hf—O3	2.1933 (13)	C10—F5	1.335 (2)
O1—C2	1.253 (2)	C10—F6	1.339 (2)
O2—C4	1.280 (2)	C11—C12	1.504 (4)
C1—C2	1.494 (3)	C11—H11A	0.96
C1—H1A	0.96	C11—H11B	0.96
C1—H1B	0.96	C11—H11C	0.96
C1—H1C	0.96	C12—C17	1.377 (4)
C2—C3	1.418 (3)	C12—C13	1.382 (4)
C3—C4	1.359 (3)	C13—C14	1.386 (4)
C3—H3	0.93	C13—H13	0.93
C4—C5	1.530 (3)	C14—C15	1.375 (4)
C5—F1	1.325 (3)	C14—H14	0.93
C5—F3	1.329 (3)	C15—C16	1.372 (4)
C5—F2	1.333 (2)	C15—H15	0.93
O3—C7	1.254 (2)	C16—C17	1.374 (4)
O4—C9	1.276 (2)	C16—H16	0.93
C6—C7	1.496 (3)	C17—H17	0.93
C6—H6A	0.96		
			
O2—Hf—O2^i^	141.66 (7)	F3—C5—C4	111.30 (17)
O2—Hf—O4	80.96 (5)	F2—C5—C4	112.58 (17)
O2^i^—Hf—O4	111.77 (5)	C7—O3—Hf	134.87 (13)
O2—Hf—O4^i^	111.77 (5)	C9—O4—Hf	131.43 (13)
O2^i^—Hf—O4^i^	80.96 (5)	C7—C6—H6A	109.5
O4—Hf—O4^i^	142.02 (7)	C7—C6—H6B	109.5
O2—Hf—O1^i^	141.35 (5)	H6A—C6—H6B	109.5
O2^i^—Hf—O1^i^	75.69 (5)	C7—C6—H6C	109.5
O4—Hf—O1^i^	72.52 (5)	H6A—C6—H6C	109.5
O4^i^—Hf—O1^i^	76.79 (5)	H6B—C6—H6C	109.5
O2—Hf—O1	75.69 (5)	O3—C7—C8	122.62 (18)
O2^i^—Hf—O1	141.35 (5)	O3—C7—C6	118.27 (18)
O4—Hf—O1	76.79 (5)	C8—C7—C6	119.06 (18)
O4^i^—Hf—O1	72.52 (5)	C9—C8—C7	120.28 (18)
O1^i^—Hf—O1	71.28 (7)	C9—C8—H8	119.9
O2—Hf—O3^i^	72.21 (5)	C7—C8—H8	119.9
O2^i^—Hf—O3^i^	76.82 (5)	O4—C9—C8	128.26 (18)
O4—Hf—O3^i^	141.11 (5)	O4—C9—C10	112.50 (17)
O4^i^—Hf—O3^i^	75.54 (5)	C8—C9—C10	119.16 (17)
O1^i^—Hf—O3^i^	143.48 (5)	F4—C10—F5	107.15 (16)
O1—Hf—O3^i^	121.11 (5)	F4—C10—F6	106.83 (17)
O2—Hf—O3	76.82 (5)	F5—C10—F6	106.94 (17)
O2^i^—Hf—O3	72.21 (5)	F4—C10—C9	111.58 (16)
O4—Hf—O3	75.54 (5)	F5—C10—C9	113.11 (17)
O4^i^—Hf—O3	141.11 (5)	F6—C10—C9	110.90 (16)
O1^i^—Hf—O3	121.11 (5)	C12—C11—H11A	109.5
O1—Hf—O3	143.48 (5)	C12—C11—H11B	109.5
O3^i^—Hf—O3	71.35 (7)	H11A—C11—H11B	109.5
C2—O1—Hf	134.34 (13)	C12—C11—H11C	109.5
C4—O2—Hf	131.45 (13)	H11A—C11—H11C	109.5
C2—C1—H1A	109.5	H11B—C11—H11C	109.5
C2—C1—H1B	109.5	C17—C12—C13	117.8 (3)
H1A—C1—H1B	109.5	C17—C12—C11	119.9 (3)
C2—C1—H1C	109.5	C13—C12—C11	122.3 (3)
H1A—C1—H1C	109.5	C12—C13—C14	120.7 (2)
H1B—C1—H1C	109.5	C12—C13—H13	119.6
O1—C2—C3	122.69 (19)	C14—C13—H13	119.6
O1—C2—C1	118.14 (18)	C15—C14—C13	120.5 (2)
C3—C2—C1	119.12 (18)	C15—C14—H14	119.8
C4—C3—C2	120.47 (18)	C13—C14—H14	119.8
C4—C3—H3	119.8	C16—C15—C14	118.9 (3)
C2—C3—H3	119.8	C16—C15—H15	120.5
O2—C4—C3	127.96 (18)	C14—C15—H15	120.5
O2—C4—C5	112.58 (17)	C15—C16—C17	120.5 (3)
C3—C4—C5	119.46 (17)	C15—C16—H16	119.8
F1—C5—F3	106.71 (18)	C17—C16—H16	119.8
F1—C5—F2	107.83 (17)	C16—C17—C12	121.6 (2)
F3—C5—F2	106.67 (17)	C16—C17—H17	119.2
F1—C5—C4	111.44 (17)	C12—C17—H17	119.2

Symmetry codes: (i) −*x*+1, *y*, −*z*+3/2.

### Hydrogen-bond geometry (Å, °)

**Table:**

*D*—H···*A*	*D*—H	H···*A*	*D*···*A*	*D*—H···*A*
C3—H3···F2	0.93	2.37	2.712 (2)	102
C8—H8···F5	0.93	2.37	2.721 (2)	102
